# Potential of *Lanistes varicus* in limiting the population of *Bulinus truncatus*

**DOI:** 10.1186/s13104-017-2837-9

**Published:** 2017-10-25

**Authors:** Francis Anto, Langbong Bimi

**Affiliations:** 1 School of Public Health, University of Ghana, Legon, Ghana; 2 Department of Animal Biology and Conservation Science, University of Ghana, Legon, Ghana

**Keywords:** *Lanistes varicus*, *Bulinus truncatus*, *Schistosoma haematobium*, Tono irrigation system, Biological control

## Abstract

**Objective:**

To determine the ability of the Ampullariid, *Lanistes varicus* to prey on egg masses and juveniles of *Bulinus truncatus* snails, an intermediate host of urogenital schistosomiasis in West Africa.

**Results:**

*Lanistes varicus* was found to feed voraciously on egg masses and juveniles of *Bulinus truncatus*, consuming all egg masses (20 –25) exposed to it within 24 h. Also, 95–100% of 1–2 days old *B. truncatus* snails exposed to a single *L. varicus* snail was consumed within 4 days. The presence of *L. varicus* snails greatly increased mortality in *B. truncatus* with mortality increasing with increase in the number of *L. varicus* snails in the mixture of the two snail species. The current study has demonstrated under laboratory conditions that the Ghanaian strain of *L. varicus* has the potential of limiting the population of *B. truncatus* snails, and contribute to the control of urogenital schistosomiasis in West Africa.

**Electronic supplementary material:**

The online version of this article (doi:10.1186/s13104-017-2837-9) contains supplementary material, which is available to authorized users.

## Introduction

Human schistosomiasis, also known as bilharzia, is a complex of acute and chronic parasitic infections caused by mammalian blood flukes of the genus *Schistosoma.* Control of the disease currently focuses on reducing morbidity through periodic, large-scale population treatment with praziquantel. It is estimated that at least 90% of those requiring treatment for schistosomiasis live in Africa [1]. School-aged children through certain childhood activities such as helping parents on irrigated farms and swimming in infested water are especially vulnerable to schistosomiasis infection [2].

Though chemotherapy reduces transmission, it rarely, if ever eliminates it in endemic populations and reinfection to pre-treatment levels can occur quickly. A more comprehensive approach including potable water, adequate sanitation and snail control is required to make impact. The molluscan family Ampullariidae, to which *Lanistes varicus* belongs, has been considered for many years as the most promising biological control agents for schistosomiasis intermediate host snails [3]. Our earlier studies have also demonstrated that *L. varicus* has the potential to control the intermediate host snail of intestinal schistosomiasis—*Biomphalaria pfeifferi* [4] which is of a more global interest. The current study was a follow-up to investigate whether *L. varicus* snail has any biological control impact on *B. truncatus* the intermediate host snail of urogenital schistosomiasis, the more common form of the disease in school-aged children in the study area [5].

## Main text

### Methods

#### Maintenance of *B. truncatus* snails and production of egg masses and juveniles

*Bulinus truncatus* snails were collected from the Tono irrigation canals in the Kassena-Nankana district of northern Ghana (10°45′ N and 1° W) and used to establish breeding colonies in the laboratory. The snails were bred in 20-L plastic aquaria using sieved water (mesh size 70 microns) collected from the canals and fed on sun-dried lettuce (*Launaea taraxacifolia*). The water was changed weekly, and the field collected adult snails removed when 50 or more egg masses were deposited. The eggs were allowed to hatch and the first generation of laboratory bred snails maintained to produce egg masses that were utilized in experiments to determine the ability of *Lanistes varicus* to feed on egg masses of *B. truncatus*. Some egg masses were allowed to hatch and the juvenile (1–2 days old) snails exposed to *L. varicus* snails to investigate its predatory potential.

#### Maintenance of *Lanistes varicus* snails

Wild *L. varicus* snails were also collected from the irrigation canals and maintained in the laboratory as described for *B. truncatus* snails. The average live weight of the *L. varicus* snails was 11.6 g. The *Lanistes* snails were used for the experiments after a period of at least 7 days acclimatisation in the laboratory. No eggs were deposited by the *Lanistes* snails during the period.

#### Experiment 1. The potential of *L. varicus* to limit the population of *B*. *truncatus* snails

Varying numbers of 5 weeks old *B. truncatus* snails were placed in separate experimental aquaria together with different numbers of *L. varicus* snails to make up a total of 20 snails per aquarium (Additional file 1). Each experimental combination of snails (denoted herein as treatment 1, 2, 3, 4 and 5) had one duplicate, there were also two controls consisting of only *B. truncatus* snails (no *L. varicus* snails). The experiment was set up and monitored for a period of 12 weeks. The number of *B. truncatus* egg masses and dead snails were recorded weekly. *Bulinus truncatus* egg masses in the control aquaria were removed weekly to avoid overcrowding.

#### Experiment 2. Consumption of *B.* truncatus egg masses and juvenile snails by *L*. *varicus*

Adult *B. truncatus* snails were kept in four different transparent plastic aquaria of volume 4 L and allowed to deposit 20–25 egg masses. The snails were then removed, the water changed and the positions of the egg masses marked on the outside of the aquaria using a marker pen. One *L. varicus* snail was then introduced into each of the aquaria and monitored for egg consumption over a period of 24-h. In another experiment, four different aquaria each containing twenty 1 to 2-day old *B. truncatus* snails and 1 adult *L. varicus* snail were set up. Consumption of these 1 to 2-day old *B. truncatus* snails by *L. varicus* was monitored daily for 4 days. Dried lettuce feed was provided ad libitum.

#### Experiment 3. Survival and growth of *B. truncatus* in the presence of *L. varicus*

Various numbers (20, 5, 10, and 15) of juvenile *B. truncatus* snails (14 days old) were placed in separate plastic aquaria and different numbers (0, 15, 10 and 5) of wild laboratory acclimatized *L. varicus* snails were added to make up a total of 20 snails per aquarium. Each combination of *B. truncatus* and *L. varicus* snails (denoted as treatment 1, 2, 3 and 4) had 2 set ups. There were also controls consisting of only *B. truncatus* snails (20 in number). The experiment was set up for 10 weeks during which feed (sun-dried lettuce) was provided ad libitum. Dead snails were removed but not replaced. At the end of each week, the *B. truncatus* snails were weighed using an electronic balance (Mettler PJ3600, Delta Range) and the live weights recorded. Free water on the snails was removed by blotting for 1–2 min before weighing.

#### Statistical analysis

Demographic variables of *B. truncatus* were compared between groups by Analysis of Variance (ANOVA) and a p value of 0.05 being taken as indicative of a statistically significant difference.

### Results

#### Experiment 1

In treatment 2 involving 30 *L. varicus* and 10 *B. truncatus* snails (30Lv:10Bt), only two *B. truncatus* egg masses were found deposited in the aquaria compared with 724 egg masses in the control (treatment 1) at the end of week 1 (Table 1). The trend persisted throughout the subsequent eleven weeks of observation. For most of the time (8/12), no egg mass was found in the aquaria of this treatment group. Only six (6) egg masses were recorded during the 12-week period giving an average of less than one egg mass per snail. In the control aquaria however, as many as 6933 *B. truncatus* egg masses were recorded during the experimental period giving an average of 15.3 egg masses per snail.

**Table 1:** Egg mass deposition by *B. truncatus* in the presence of *L. varicus* snails

Week	Treatment 1		Treatment 2		Treatment 3		Treatment 4	
	No. of live *B. truncatus* snails	No. of egg masses (density)^a^	No. of live Lv:Bt	No. of egg masses (density)^a^	No. of live Lv:Bt	No. of egg masses (density)^a^	No. of live Lv:Bt	No. of egg masses (density)^a^
1	40	724 (18.1)	30:10	2 (0.2)	20:20	3 (0.2)	10:30	2 (0.2)
2	40	647 (16.2)	30:10	0 (0.0)	20:20	3 (0.2)	10:28	4 (0.5)
3	40	819 (20.5)	30:10	0 (0.0)	20:20	3 (0.2)	10:28	4 (0.1)
4	38	695 (18.3)	29:9	1 (0.1)	20:19	0 (0.0)	10: 28	4 (0.2)
5	38	611 (16.1)	29:8	1 (0.1)	20:19	1 (0.1)	10:26	7 (0.3)
6	38	652 (17.2)	29:8	0 (0.0)	20:19	0 (0.0)	10:26	3 (0.3)
7	38	595 (15.7)	29:7	0 (0.0)	20:15	2 (0.1)	10:26	2 (0.1)
8	38	407 (10.7)	29:7	2 (0.3)	20:13	8 (0.6)	10:26	1 (0.3)
9	37	462 (12.5)	28:5	0 (0.0)	20:10	0 (0.0)	10:24	0 (0.4)
10	37	418 (11.3)	28:5	0 (0.0)	20:8	2 (0.3)	10:23	9 (0.3)
11	35	483 (13.8)	28:3	0 (0.0)	20:7	0 (0.0)	10:20	84 (4.2)
12	34	420 (12.4)	28:3	0 (0.0)	20:7	2 (0.3)	10:20	13 (0.7)
Total	453	6933 (*15.3*)	347:85	6 (0.7)	240:177	24 (1.8)	100:305	133 (6.2)

*Lv:Bt* number of *L. varicus*:number of *B. truncatus* snails

^a^Average number of egg masses/snail

The experiments involving 20 *L. varicus*/20 *B. truncatus* (treatment 3) and 10 *L. varicus*/30 *B. truncatus* snails (treatment 4) also gave similar results as the one described for treatment 2, with significantly more (*p* < 0.001) egg masses per snail in the control than in treatments 3 and 4 (Table 2).

**Table 2:** Comparison of number of egg masses deposited by experimental groups

Groups	Sum of squares (SS)	Degrees of freedom (df)	Mean of squares (MS)	F-statistic	p value
Between groups	2,958,040.5	3	986,013.5	203.69	< 0.001
Within groups	212,996.167	44	4840.821		
Total	3,171,036.67	47	67,468.865		

Treatment	Contrast	Standard error	t-statistic		p value
2 vs 1	− 577.25	28.40	− 20.32		< 0.001
3 vs 1	− 575.75	28.40	− 20.27		< 0.001
4 vs 1	− 566.67	28.40	− 19.95		< 0.001
3 vs 2	1.5	28.40	0.05		1.000
4 vs 2	10.58	28.40	0.37		0.982
4 vs 3	9.08	28.40	0.32		0.989

Among treatments 2, 3 and 4, there were variations in the number of egg masses recorded per week. Overall, a total of 72 egg masses were recorded for these treatment groups during the 12-week period. The least number, 6 (8.3%) of egg masses was recorded in treatment 2 and the highest in treatment 4 but the differences were not statistically significant (p > 0.05) (Table 2).

#### Experiment 2

A single *L. varicus* snail was able to consume all (20–25) *B. truncatus* egg masses exposed to it within 24 h. Also a single *L. varicus* snail was able to prey on 95–100% of juvenile snails exposed to it within 4 days of observation (Additional file 2).

#### Experiment 3

**Fig. 1:**
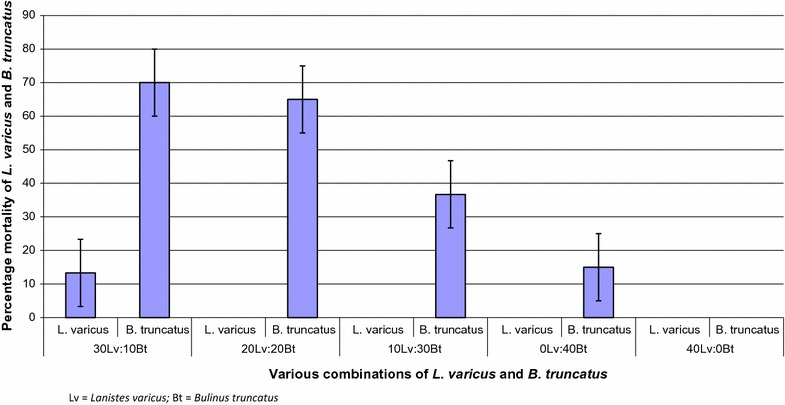
Mortality of *L. varicus* and *B. truncatus* in mixed cultures of the two snail species. Lv, *Lanistes varicus*; Bt, *Bulinus truncatus*

Mortality of *B. truncatus* snails (6/40, 15%) in treatment 1 (control) was significantly lower (p < 0.002) than in treatment 2 (7/10, 70%) at the end of the 10-week experimental period. Similarly, the difference in mortality (13/20, 65%) between Treatment 3 (20Lv:20Bt) and the control was highly significant (p < 0.001). Mortality of *B. truncatus* snails in treatment 4 (10/30, 33%) was however not significantly higher (p > 0.05) than in the control. Mortality of *L. varicus* snails in the mixture was very low as only in Treatment 2 that mortality of *L. varicus* snail occurred (Fig. 1).

The presence of *L. varicus* snails in mixtures of the Ampullariid and *B. truncatus* did not significantly affect the growth rate of *B. truncatus*. At the start of the experiment, *B. truncatus* snails in treatment 2 had the same mean live weight as those in the control group (0.02 g). At the end of the experimental period, each *B. truncatus* snail in treatment 2 still had equal mean live weight (0.1 g) as those in the control. Results of treatments 3 and 4 were similar to those described for treatment 2 with no detectable difference in live weight between *B. truncatus* snails in the treatment and the control groups (weight measurement data available as Additional file 3).

### Discussion

The ability of *Lanistes varicus* snail to prey on egg masses and juvenile snails of *B. truncatus* was investigated with the aim of determining its biocontrol potential under laboratory conditions that could be exploited for the control of urogenital schistosomiasis in West Africa.

Very low numbers of *B. truncatus* egg masses were recorded for all the experiments in which both *B. truncatus* and *L. varicus* were bred together. These results suggest that the presence of *L. varicus* snails in the mixture affected the *B. truncatus* egg count either through the secretion of substances into the aquarium [6] or through competitive interactions especially for food or that *B. truncatus* did deposit egg masses at a normal rate but were removed from the aquaria by *L. varicus* snails through direct predation on the egg masses [7, 8].

Predation on *B. truncatus* egg masses by *L. varicus* snails is supported by results from “Experiment 2”, in which all (20–25) *B. truncatus* egg masses exposed to a single *L. varicus* snail were consumed within 24 h. Similarly, *L. varicus* snails were observed to have consumed 95–100% of juvenile *B. truncatus* snails made available within 4 days. These observations imply that, *L. varicus* snails from Ghana are voracious predators [8] of both egg masses and juvenile snails of *B. truncatus* [9]. Starvation is not a likely reason for the low egg count in the mixed cultures as feed in the form of dried lettuce was provided *al libitum* [10].

In addition to *L. varicus* preying on the egg masses and juveniles of *B. truncatus* snails, it increased significantly the mortality of the schistosome intermediate host snails. There was a relationship between the *L. varicus/B. truncatus* ratio [7]; the higher the number of *L. varicus* snails in the mixture, the higher the level of mortality among the *B. truncatus* snails. The *L. varicus* snails in the current work could be interacting with *B. truncatus* in a way detrimental to the survival of the schistosomiasis intermediate host; possibly as a result of release of chemical substances into the mixture.

The current study has demonstrated the potential of *L. varicus* snail as a biocontrol agent of *B. truncatus. Lanistes varicus* is a voracious predator on *B. truncatus* and is likely to treat the intermediate host snail as highly preferred food. Therefore, further research in the context of biological control of schistosomiasis is warranted. Good results in such experiments could provide a relatively cheap and sustainable option for controlling schistosomiasis in Ghana and other countries in the sub-region including Burkina Faso, Mali and Niger where *B. truncatus* transmits *Schistosoma haematobium,* the causative agent of urogenital schistosomiasis.

## Limitations

The main limitations of the study are:

1. The juvenile *B. truncatus* snails exposed to *L. varicus* may be too young, small and fragile.
2. The actual predation of *L. varicus* on the juvenile *B. truncatus* snails could not be observed as we were unable to stay overnight to observe the activities of the snails.
3. The relatively larger *L. varicus* snails could have crushed some of the tiny (1–2 days old) *B. trucatus* snails by just crawling over them and not actually feeding on all the exposed snails.

## Additional files

**Additional file 1.** Arrangement of *B. truncatus and L. varicus* in snail population control experiments.

**Additional file 2.** Percentage of *B. truncatus* egg masses or juvenile snails consumed by a single adult *L. varicus* snail.

**Additional file 3.** Weight measurement data.

## Abbreviations

*B. truncatus*: *Bulinus truncatus*
*L. varicus*: *Lanistes varicus*
Lv:Bt: Number of *Lanistes varicus:Bulinus truncatus*
S.E: standard error
