# Supplementary material for: Potential of Lanistes varicus in limiting the population of Bulinus truncatus
Source: BMC Res Notes. 2017 Oct 25;10:509. doi: 10.1186/s13104-017-2837-9 (PMC5657124; doi:10.1186/s13104-017-2837-9)
Supplement: Supplementary file 1 — Additional file 1. Arrangement of B. truncatus and L. varicus in snail population control experiments. [file 13104_2017_2837_MOESM1_ESM.docx]

**Additional file 1: Arrangement of *B. truncatus and L. varicus* in snail population control experiments**

| Treatment | Number of experimental  snails per aquarium | | Total number of snails per treatment group | | Number of replicates |
| --- | --- | --- | --- | --- | --- |
|  | *L. varicus* | *B. truncatus* | *L. varicus* | *B. truncatus* |  |
| 1^a^ | 0 | 20 (control) | 0 | 40 | 2 |
| 2 | 15 | 5 | 30 | 10 | 2 |
| 3 | 10 | 10 | 20 | 20 | 2 |
| 4 | 5 | 15 | 10 | 30 | 2 |
| 5^b^ | 20 (control) | 0 | 40 | 0 | 2 |

^a^ ^Control experiment consisting of only^ *^B. truncatus^* ^snails.^

^b Control experiment consisting of only^ *^L. varicus^* ^snails.^
