# Supplementary material for: Potential of Lanistes varicus in limiting the population of Bulinus truncatus
Source: BMC Res Notes. 2017 Oct 25;10:509. doi: 10.1186/s13104-017-2837-9 (PMC5657124; doi:10.1186/s13104-017-2837-9)
Supplement: Supplementary file 2 — Additional file 2. Percentage of B. truncatus egg masses or juvenile snails consumed by a single adult L. varicus snail. [file 13104_2017_2837_MOESM2_ESM.docx]

**Additional file 2: Percentage of *B. truncatus* egg masses or juvenile snails consumed by a single adult *L.* *varicus* snail**

| Stage of *B. truncatus* | Duration of | % consumed (± S.E.) |
| --- | --- | --- |
| exposed | exposure |  |
| Egg masses | 24 hrs | 100 (± 0.0) |
| Juvenile snails | 4 days | 97.5 (± 1.4) |
