# Supplementary material for: Potential of Lanistes varicus in limiting the population of Bulinus truncatus
Source: BMC Res Notes. 2017 Oct 25;10:509. doi: 10.1186/s13104-017-2837-9 (PMC5657124; doi:10.1186/s13104-017-2837-9)
Supplement: Supplementary file 3 — Additional file 3. Weight measurement data. [file 13104_2017_2837_MOESM3_ESM.docx]

**Additional file 3a. Survival and growth rate of *B. truncatus* in the presence of *L. varicus* (ratio = 15 *L. varicus* to 5 *B. truncates*)**

| Period | No. of live *B. truncatus* snails in control and | | | | | | | | Weight of *B. truncatus* snails in control | | | | | | | Control | Treatment |
| --- | --- | --- | --- | --- | --- | --- | --- | --- | --- | --- | --- | --- | --- | --- | --- | --- | --- |
| (wks) | treatment aquaria | | | | | | |  | and treatment aquaria | | | | | | | weight (g) | weight (g) |
|  | Control aquaria | | |  | Treatment aquaria | | |  | Control aquaria | | |  | Treatment aquaria | | | per | per |
|  | Aq 1 | Aq 2 | Total | | Aq 1 | Aq 2 | Total | | Aq 1 | Aq 2 | Total | | Aq 1 | Aq 2 | Total | snail | Snail |
| 0 | 20 | 20 | 40 | | 5 | 5 | 10 | | 0.32 | 0.31 | 0.63 | | 0.1 | 0.1 | 0.2 | 0.016 | 0.020 |
| 1 | 19 | 20 | 39 | | 5 | 5 | 10 | | 0.84 | 0.89 | 1.73 | | 0.21 | 0.21 | 0.42 | 0.044 | 0.042 |
| 2 | 19 | 20 | 39 | | 5 | 5 | 10 | | 1.12 | 1.19 | 2.31 | | 0.27 | 0.24 | 0.51 | 0.059 | 0.051 |
| 3 | 19 | 19 | 38 | | 5 | 5 | 10 | | 1.37 | 1.48 | 2.85 | | 0.33 | 0.28 | 0.61 | 0.075 | 0.061 |
| 4 | 18 | 19 | 37 | | 5 | 5 | 10 | | 1.69 | 1.99 | 3.68 | | 0.39 | 0.34 | 0.73 | 0.099 | 0.073 |
| 5 | 18 | 18 | 36 | | 5 | 4 | 9 | | 1.86 | 2.17 | 4.03 | | 0.42 | 0.3 | 0.72 | 0.112 | 0.080 |
| 6 | 17 | 18 | 35 | | 5 | 4 | 9 | | 1.99 | 2.35 | 4.34 | | 0.45 | 0.33 | 0.78 | 0.124 | 0.087 |
| 7 | 17 | 18 | 35 | | 5 | 4 | 9 | | 2.12 | 2.52 | 4.64 | | 0.48 | 0.37 | 0.85 | 0.133 | 0.094 |
| 8 | 16 | 17 | 33 | | 5 | 4 | 9 | | 1.95 | 2.38 | 4.33 | | 0.5 | 0.4 | 0.9 | 0.131 | 0.100 |
| 9 | 16 | 17 | 33 | | 4 | 2 | 6 | | 2.13 | 2.47 | 4.6 | | 0.45 | 0.17 | 0.62 | 0.139 | 0.103 |
| 10 | 16 | 16 | 32 | | 3 | 0 | 3 | | 2.2 | 2.45 | 4.65 | | 0.31 | ----- | 0.31 | 0.145 | 0.103 |
| Total | 195 | 202 | 397 | | 52 | 43 | 95 | | 17.59 | 20.2 | 37.79 | | 3.91 | 2.74 | 6.65 | 1.078 | 0.815 |

^wks = weeks; Aq 1 = aquarium 1; Aq 2 = aquarium 2.^

**Additional file 3b. Survival and growth rate of *B. truncatus* in the presence of *L. varicus* (ratio = 10 *L. varicus* to 10 *B. truncatus*)**

| Period | No. of live *B. truncatus* snails in control and | | | | | | | | Weight of *B. truncatus* in control | | | | | | | Control | Treatment |
| --- | --- | --- | --- | --- | --- | --- | --- | --- | --- | --- | --- | --- | --- | --- | --- | --- | --- |
| (wks) | treatment aquaria | | | | | | |  | and treatment aquaria | | | | | | | weight (g) | weight (g) |
|  | Control aquaria | | |  | Treatment aquaria | | |  | Control aquaria | | |  | Treatment aquaria | | | per | per |
|  | Aq 1 | Aq 2 | Total | | Aq 1 | Aq 2 | Total | | Aq 1 | Aq 2 | Total | | Aq 1 | Aq 2 | Total | snail | Snail |
| 0 | 20 | 20 | 40 | | 10 | 10 | 20 | | 0.32 | 0.31 | 0.63 | | 0.17 | 0.14 | 0.31 | 0.016 | 0.016 |
| 1 | 19 | 20 | 39 | | 10 | 7 | 17 | | 0.84 | 0.89 | 1.73 | | 0.4 | 0.26 | 0.66 | 0.044 | 0.039 |
| 2 | 19 | 20 | 39 | | 10 | 7 | 17 | | 1.12 | 1.19 | 2.31 | | 0.62 | 0.35 | 0.97 | 0.059 | 0.057 |
| 3 | 19 | 19 | 38 | | 10 | 7 | 17 | | 1.37 | 1.48 | 2.85 | | 0.75 | 0.42 | 1.17 | 0.075 | 0.069 |
| 4 | 18 | 19 | 37 | | 10 | 7 | 17 | | 1.69 | 1.99 | 3.68 | | 0.86 | 0.53 | 1.39 | 0.099 | 0.082 |
| 5 | 18 | 18 | 36 | | 10 | 6 | 16 | | 1.86 | 2.17 | 4.03 | | 0.9 | 0.51 | 1.41 | 0.112 | 0.088 |
| 6 | 17 | 18 | 35 | | 9 | 5 | 14 | | 1.99 | 2.35 | 4.34 | | 0.93 | 0.51 | 1.44 | 0.124 | 0.103 |
| 7 | 17 | 18 | 35 | | 8 | 5 | 13 | | 2.12 | 2.52 | 4.64 | | 0.9 | 0.56 | 1.46 | 0.133 | 0.112 |
| 8 | 16 | 17 | 33 | | 6 | 5 | 11 | | 1.95 | 2.38 | 4.33 | | 0.7 | 0.58 | 1.28 | 0.131 | 0.116 |
| 9 | 16 | 17 | 33 | | 5 | 5 | 10 | | 2.13 | 2.47 | 4.6 | | 0.56 | 0.65 | 1.21 | 0.139 | 0.121 |
| 10 | 16 | 16 | 32 | | 2 | 5 | 7 | | 2.2 | 2.45 | 4.65 | | 0.22 | 0.67 | 0.89 | 0.145 | 0.127 |
| Total | 195 | 202 | 397 | | 90 | 69 | 159 | | 17.59 | 20.2 | 37.79 | | 7.01 | 5.18 | 12.19 | 1.078 | 0.930 |

^wks = weeks; Aq 1 = aquarium 1; Aq 2 = aquarium 2.^

**Additional file 3c. Survival and growth rate of *B. truncatus* in the presence of *L. varicus* (ratio = 5 *L. varicus* to 15 *B. truncatus*)**

| Period | No. of live *B. truncatus* snails in control and | | | | | | | | Weight of *B. truncatus* snails in control | | | | | | | Control | Treatment |
| --- | --- | --- | --- | --- | --- | --- | --- | --- | --- | --- | --- | --- | --- | --- | --- | --- | --- |
| (wks) | treatment aquaria | | | | | | |  | and treatment aquaria | | | | | | | weight (g) | weight (g) |
|  | Control aquaria | | |  | Treatment aquaria | | |  | Control aquaria | | |  | Treatment aquaria | | | per | per |
|  | Aq 1 | Aq 2 | Total | | Aq 1 | Aq 2 | Total | | Aq 1 | Aq 2 | Total | | Aq 1 | Aq 2 | Total | snail | Snail |
| 0 | 20 | 20 | 40 | | 15 | 15 | 30 | | 0.32 | 0.31 | 0.63 | | 0.23 | 0.24 | 0.47 | 0.016 | 0.016 |
| 1 | 19 | 20 | 39 | | 14 | 14 | 28 | | 0.84 | 0.89 | 1.73 | | 0.55 | 0.55 | 1.1 | 0.044 | 0.039 |
| 2 | 19 | 20 | 39 | | 14 | 14 | 28 | | 1.12 | 1.19 | 2.31 | | 0.72 | 0.69 | 1.41 | 0.059 | 0.050 |
| 3 | 19 | 19 | 38 | | 14 | 14 | 28 | | 1.37 | 1.48 | 2.85 | | 0.87 | 0.83 | 1.7 | 0.075 | 0.061 |
| 4 | 18 | 19 | 37 | | 14 | 14 | 28 | | 1.69 | 1.99 | 3.68 | | 1.07 | 0.96 | 2.03 | 0.099 | 0.073 |
| 5 | 18 | 18 | 36 | | 14 | 14 | 28 | | 1.86 | 2.17 | 4.03 | | 1.14 | 1.04 | 2.18 | 0.112 | 0.078 |
| 6 | 17 | 18 | 35 | | 14 | 14 | 28 | | 1.99 | 2.35 | 4.34 | | 1.3 | 1.16 | 2.46 | 0.124 | 0.088 |
| 7 | 17 | 18 | 35 | | 14 | 14 | 28 | | 2.12 | 2.52 | 4.64 | | 1.37 | 1.29 | 2.66 | 0.133 | 0.095 |
| 8 | 16 | 17 | 33 | | 14 | 14 | 28 | | 1.95 | 2.38 | 4.33 | | 1.4 | 1.36 | 2.76 | 0.131 | 0.099 |
| 9 | 16 | 17 | 33 | | 14 | 14 | 28 | | 2.13 | 2.47 | 4.6 | | 1.43 | 1.4 | 2.83 | 0.139 | 0.101 |
| 10 | 16 | 16 | 32 | | 13 | 13 | 26 | | 2.2 | 2.45 | 4.65 | | 1.36 | 1.34 | 2.7 | 0.145 | 0.104 |
| Total | 195 | 202 | 397 | | 154 | 154 | 308 | | 17.59 | 20.2 | 37.79 | | 11.44 | 10.86 | 22.3 | 1.078 | 0.803 |

^wks = weeks; Aq 1 = aquarium 1; Aq 2 = aquarium 2.^
